# TSPO ligand residence time: a new parameter to predict compound neurosteroidogenic efficacy

**DOI:** 10.1038/srep18164

**Published:** 2016-01-11

**Authors:** Barbara Costa, Eleonora Da Pozzo, Chiara Giacomelli, Elisabetta Barresi, Sabrina Taliani, Federico Da Settimo, Claudia Martini

**Affiliations:** 1 Department of Pharmacy, University of Pisa, via Bonanno, 6-56126 Pisa, Italy

## Abstract

The pharmacological activation of the cholesterol-binding Translocator Protein (TSPO) leads to an increase of endogenous steroids and neurosteroids determining benefic pleiotropic effects in several pathological conditions, including anxiety disorders. The relatively poor relationship between TSPO ligand binding affinities and steroidogenic efficacies prompted us to investigate the time (Residence Time, RT) that a number of compounds with phenylindolylglyoxylamide structure (PIGAs) spends in contact with the target. Here, given the poor availability of TSPO ligand kinetic parameters, a kinetic radioligand binding assay was set up and validated for RT determination using a theoretical mathematical model successfully applied to other ligand-target systems. TSPO ligand RT was quantified and the obtained results showed a positive correlation between the period for which a drug interacts with TSPO and the compound ability to stimulate steroidogenesis. Specifically, the TSPO ligand RT significantly fitted both with steroidogenic efficacy (E_max_) and with area under the dose-response curve, a parameter combining drug potency and efficacy. A positive relation between RT and anxiolytic activity of three compounds was evidenced. In conclusion, RT could be a relevant parameter to predict the steroidogenic efficacy and the *in vivo* anxiolytic action of new TSPO ligands.

The 18 kDa Translocator Protein (TSPO) is an outer mitochondrial membrane high affinity cholesterol- and drug-binding protein abundant in steroid-producing tissues, including gonads, adrenal, and brain^1^. Previous pharmacological, biochemical and genetic studies, as well as *in vivo* experiments, have provided several lines of evidence demonstrating that TSPO is a key member of the multiprotein complex transduceosome, as it participates to the cholesterol translocation into mitochondria, which is considered the rate-limiting step of steroidogenesis^2^.

Consistent with the important role of TSPO in steroidogenesis^3^ and the involvement of steroids in numerous fundamental processes^8^,^9^, TSPO ligands have been proposed as innovative therapeutic tools in several pathological conditions. For example, TSPO ligands have been implicated in axonal regeneration^10^,^11^,^12^, in anti-inflammatory^13^, anxiolytic^14^,^15^,^16^,^17^,^18^, antidepressant^19^ and anti-post-traumatic stress^20^ activities, both in animal models, and in individuals with neurological or psychiatric disorders. To date, phase II and III clinical trials have been concluded or are ongoing for the treatment of diabetic peripheral neuropathy (ClinicalTrials.gov identifier: NCT00502515), chemotherapy-induced peripheral neuropathy (NCT00868166), and generalized anxiety disorder (NCT00108836).

However, one of the most recurrently issue concerning TSPO ligands consists in the lack of correlation between the binding affinity and the *in vitro* efficacy, including steroidogenic efficacy^21^. This phenomenon has limited not only the identification of lead compounds during the traditional affinity-based drug discovery processes, but also questioned the specificity of the observed effects^22^,^23^,^24^. Recent studies have shown that the affinity of a ligand for its target could not directly define its biological action effectiveness, that it may instead be related to the period for which a drug interacts with its target defined as ‘Residence Time’ (RT)^25^,^26^.

In the present work, it was investigated whether the RT could be a crucial measure to estimate the steroidogenic efficacy of a TSPO ligand. To this aim, a number of our previous reported TSPO ligands, belonging to phenylindolylglyoxylamides (PIGAs) was selected based on their different abilities to stimulate *in vitro* steroidogenesis^27^,^28^,^29^, Table 1. Among such selected TSPO compounds, three presented *in vivo* anxiolytic effects^28^,^30^,^31^.

As a first step, given the poor availability of kinetic parameters for TSPO ligands, a kinetic radioligand binding assay was set up and validated for TSPO ligand RT determination by the use of the theoretical mathematical model of Motulsky and Mahan^32^(RT = 1/k_off_, where k_off_ is the dissociation rate constant). This model has been applied to several ligand-target systems and shown to be highly accurate in determining the binding kinetics of synthetic ligands^33^,^34^,^35^.

## Results

### RT determination: initial setting

The [^3^H]PK11195 radioligand was used as a probe for the determination of the kinetic parameters using membrane homogenates of rat kidney, a tissue highly rich in TSPO. Initial experiments were performed to fully characterize [^3^H]PK11195 binding kinetic parameters as few literature data were available^36^,^37^. [^3^H]PK11195 equilibrium binding parameters, K_d_ and B_max_, were 3.60 ± 0.41 nM and 6498 ± 500 fmol/mg of proteins, respectively (Table 2). The K_i_ value of unlabeled PK11195 determined by [^3^H]PK11195 displacement binding assay was 3.39 ± 0.34 nM (Table 2).

**Figure 1:**
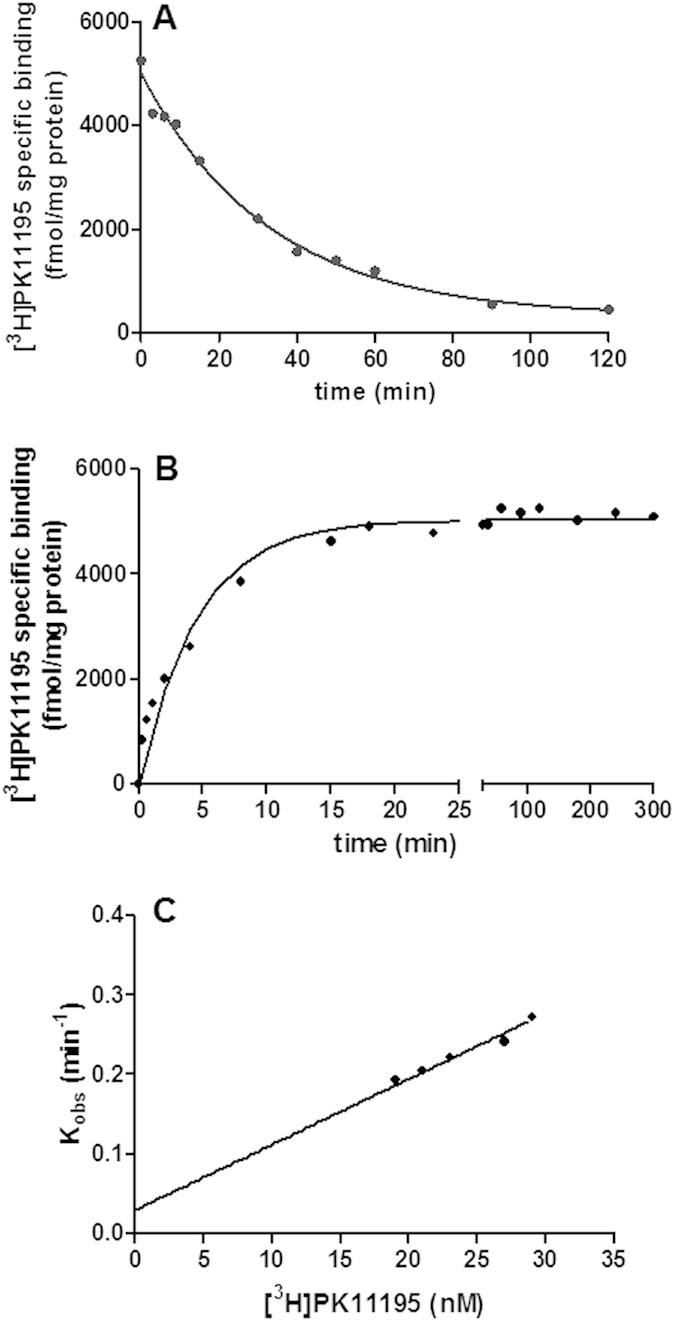
[^3^H]PK11195 k_on_ and k_off_ by ‘traditional’ dissociation and association kinetics assays. (**A**) [^*3*^*H*]*PK11195 binding dissociation Kinetics*: Data were best fitted using a one-phase exponential decay function to produce a t_1/2_ estimate. This was converted into a k_off_ value by using Equation 1, as detailed in the Materials and Methods section. The ordinate reports the specific [^3^H]PK11195 binding expressed as fmol/mg of protein. The abscissa reports the incubation time expressed in min. (**B,C**) [^*3*^*H*]*PK11195 binding association Kinetics:* a family of association kinetics curves were constructed incubating membrane homogenates with a range of [^3^H]PK11195 concentrations as described in the Materials and Methods section. Data were fitted using a one phase exponential association function to yield a k_ob_. (**B**) A representative association curve performed using 23 nM [^3^H]PK11195 up to 5 h incubation time is showed. The ordinate reports the specific [^3^H]PK11195 binding expressed as fmol/mg of protein. The abscissa reports the incubation time expressed in min. (**C**) k_obs_ values plotted against the corresponding [^3^H]PK11195 concentration employed are showed.

The k_off_ determination was obtained by: (i) pre-labelling of TSPO to equilibrium with one [^3^H]PK11195 concentration (approximately 10 × K_d_) that provides high initial TSPO occupancy; (ii) inducing radioligand dissociation by addition of TSPO-saturating concentration (about 1000 × K_d_) of unlabeled competing compound PK11195. Then, the dissociation time-course was analyzed using an exponential function. An example of [^3^H]PK11195 dissociation kinetic curve is shown in Fig. 1A; [^3^H]PK11195 dissociation was monophasic and gave a half-life of radioligand-TSPO complex (t_1/2_) of 23 min, which when applied to Equation 1 (see Materials and Methods section) gave a k_off_ of 0.030 ± 0.002 min^−1^. The RT value, which is the reciprocal of k_off_, was 33 ± 4 min (Table 2).

To determine [^3^H]PK11195 k_on_, a family of association kinetic curves using a range of radioligand concentrations were constructed. Each association curve was monitored until equilibrium. In Fig. 1B, a representative [^3^H]PK11195 association kinetics curve is showed. If [^3^H]PK11195 binding follows a simple law of mass action model, the observed kinetic association constant (k_ob_) should increase in a linear manner with radioligand concentration^38^. In this case, the slope of the line should equate to the association rate and extrapolation of the plot to the Y-intercept (at x = 0) should equal the dissociation rate^39^. When the k_ob_ values were plotted against radioligand concentration, the data were consistent with a straight line (r^2^ = 0.98), indicating that binding of [^3^H]PK11195 to TSPO was consistent with the law of mass action (Fig. 1C). The obtained values were k_on_ = 8.30 ± 0.64 × 10^6^ M^−1^ min^−1^ and k_off_ = 0.030 ± 0.002 min^−1^ (Fig. 1C and Table 2). The kinetically derived K_d_ (3.61 ± 0.21 nM; kinetic K_d_ = k_off_/k_on_) was in good agreement with the value obtained from [^3^H]PK11195 saturation experiments (equilibrium K_d_ = 3.60 ± 0.41) (Table 2).

### RT Determination: TSPO ligand k_off_ and k_on_ by competition kinetic association assays

With the predetermined k_on_ (k_1_) and k_off_ (k_2_) values of [^3^H]PK11195 from ‘traditional’ kinetic association and dissociation experiments, k_on_ (k_3_) and k_off_ (k_4_) of an unlabeled ligand could be determined by fitting the kinetic parameters into the model of ‘Kinetics of competitive binding’ described in Materials and Methods section. This method is based on a framework developed by Motulsky and Mahan^32^, where an unlabeled competitor is added simultaneously with a radioligand to the receptor preparation of interest. Then, the experimentally derived rate of specific radioligand binding can be modelled to provide the association and dissociation rates of the unlabeled compound.

**Figure 2:**
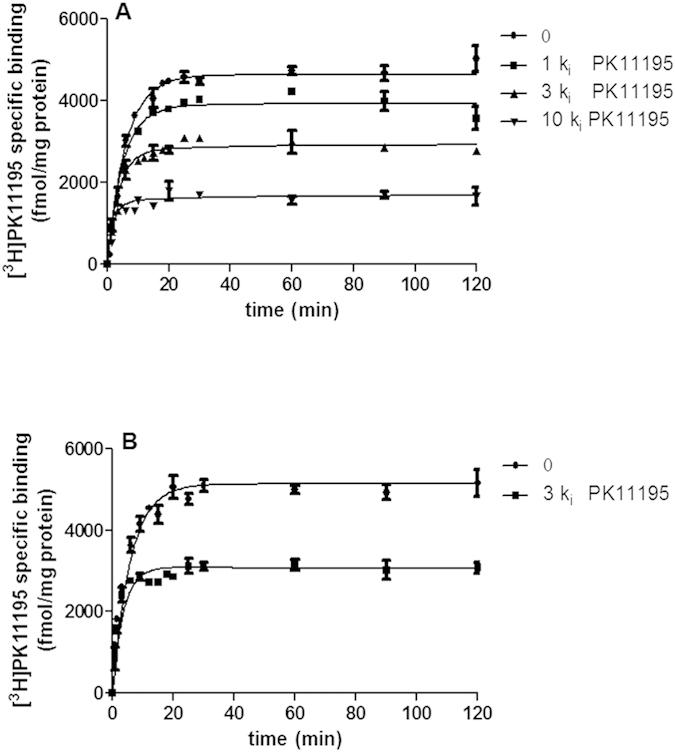
PK11195 k_on_ and k_off_ by competitive association kinetics assays. **(A)** The curves were obtained by incubation of membranes with either radioligand alone or radioligand and unlabeled PK11195 for the indicated time points. Data were fitted to the equation 2 to calculate the k_on_ and k_off_ of PK11195. Representative curves obtained using one-, three-, ten-fold K_i_ value of unlabeled PK11195. **(B)** Representative curves obtained using three-fold K_i_ value of unlabeled PK111195.

As a first step, the competition association assay was performed using unlabeled PK11195. Three different concentrations of PK11195 were tested to ensure that the rate parameters calculated were independent of ligand concentration (Fig. 2A). The k_on_ (k_3_) and k_off_ (k_4_) values determined in this assay were 9.20 ± 0.71 × 10^6^ M^−1^ min^−1^ and 0.034 ± 0.004 min^−1^, respectively (Table 2; PK11195 RT = 29 ± 3 min), which corresponded rather well to the kinetics rates determined by ‘traditional’ association and dissociation experiments (Table 2). Moreover, the kinetically derived K_d_ obtained from the competition association assay for unlabeled PK11195 was similar to K_i_ obtained from displacement experiments and K_d_ derived from saturation experiments (Table 2). Taken together, these findings proved that the competition association assay could be applied to determine the binding kinetics of an unlabeled TSPO ligand.

The competition association assay approach has been shown to be highly accurate in determining the binding kinetics at several targets^33^,^34^,^35^. However, when the kinetics of multiple compounds need to be determined, the standard model is laborious and time consuming because it implies the use of three concentrations of each unlabeled ligand. Recently, it has been demonstrated that the use of one concentration of unlabeled ligand is able to yield an accurate determination of kinetic rates of unlabeled ligands at their receptor, too^34^. These findings prompted us to modify the three-concentration-dependent assay into a one-concentration-based method. The data analyzed at three-fold K_i_ of unlabeled PK11195 showed a comparable result (k_on_ = 9.30 ± 0.94 × 10^6^ M^−1^ min^−1^ and K_off_ = 0.029 ± 0.003 min^−1^; RT = 34 ± 3 min) (Fig. 2B) to that generated in a standard (three-concentration-dependent) competition association experiment (Table 2). This result indicates that this simplified method is strong enough to quantify the binding kinetics, which eventually enables testing in a faster medium-throughput format, yet without loss of accuracy.

**Figure 3:**
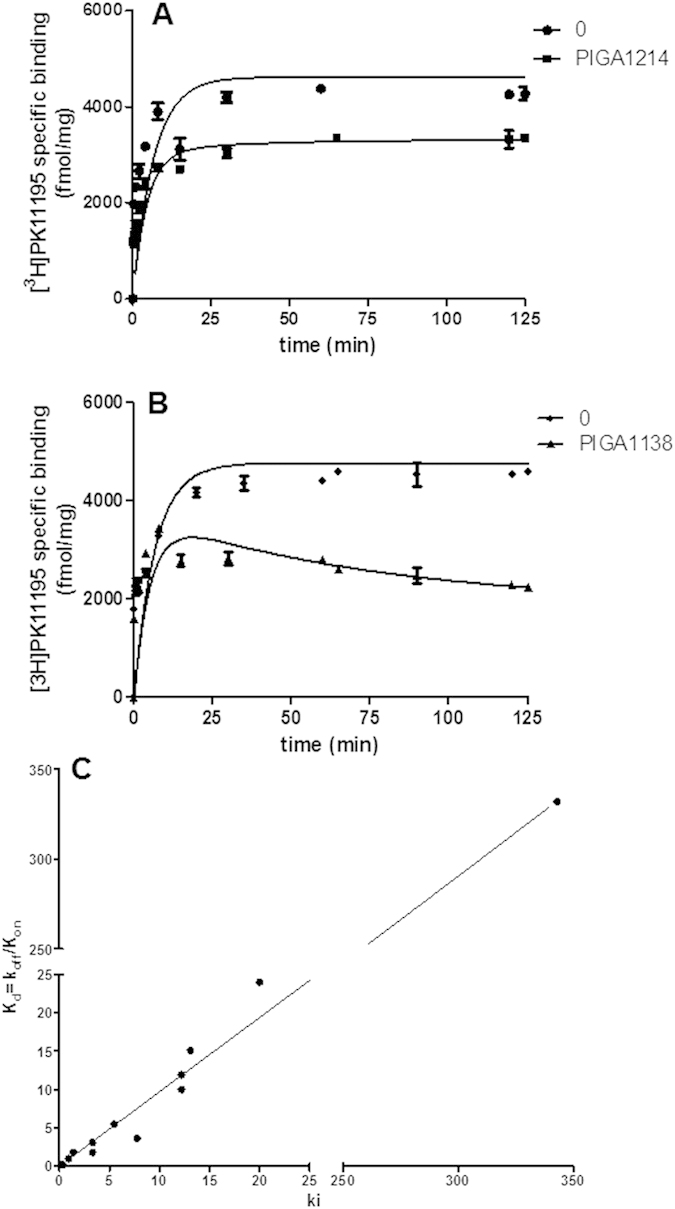
Competitive association kinetics assays of TSPO ligands and correlation between K_i_ and ‘Kinetic K_d_’ values. (**A**) Representative curves obtained using three-fold K_i_ of PIGA1214 **(B)** or PIGA1138. **(C)** K_i_ values were obtained from [^3^H]PK11195 competition binding experiments at equilibrium. The kinetically K_d_ values were derived from the competition association experiments.

By using the ‘simplified’ competition kinetic association assay, the TSPO ligands, including the classical ones Ro5-4864 and PIGA compounds (Table 1) were tested at three-fold respective K_i_ concentration and data were fitted using Equation 2 (see Material and Methods section) to calculate k_on_ and k_off_ simultaneously (Table 2). Competition association assay results demonstrated two patterns of [^3^H]PK11195 binding in dependence of the competing ligand used. In general, if the competitor dissociates from its target faster than the radioligand, the specific binding of the radioligand will approach its equilibrium time slowly and monotonically. However, when the competitor dissociates slower, the association curve of the radioligand will consist of two phases, starting with a typical “overshoot” and then a decline until a new equilibrium is reached. The results obtained are consistent with a rapid (PIGA719, PIGA720, PIGA745, PIGA835, PIGA925, PIGA1214, Ro5-4864) and a slow (PIGA823, PIGA839 (M-PIGA), PIGA1128, PIGA1138) dissociation rate of the ligands from TSPO. Representative curves for rapid dissociating (PIGA1214) and slow dissociating (PIGA1138) TSPO ligands were shown in Fig. 3A,B, respectively.

To validate the rate constants, the kinetically derived K_d_ were compared with K_i_ obtained from equilibrium competition binding experiments. Notably, an excellent correlation (r^2^ = 0.999, p < 0.0001; Fig. 3C) was observed between K_i_ determined in equilibrium-binding studies and K_d_ values derived from the competition association assays (Table 2). This further proved that the simplified model is able to quantify the association and dissociation rates of unlabeled TSPO ligands accurately.

### TSPO ligand steroidogenic efficacy

**Figure 4:**
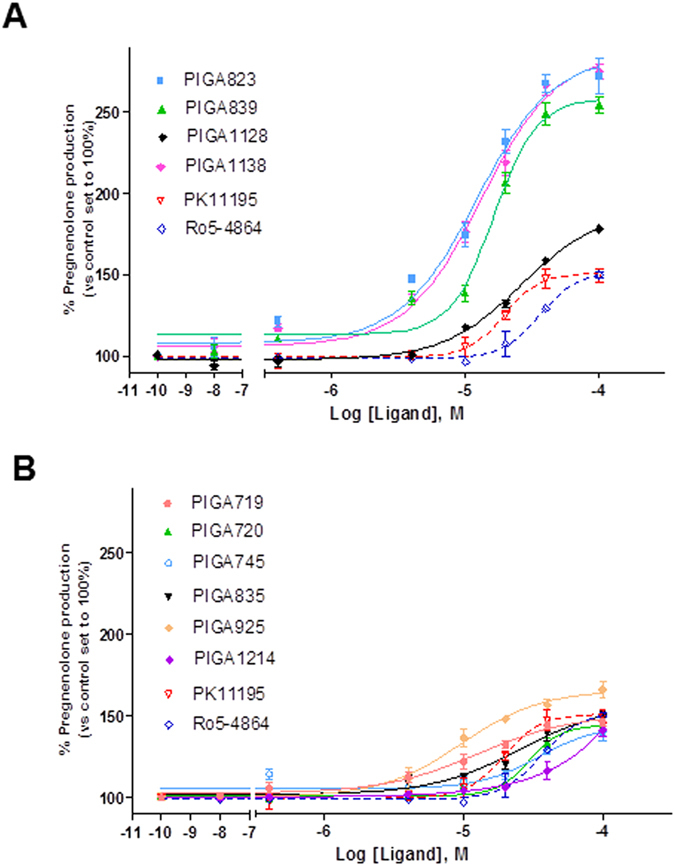
Effects of the TSPO ligands (PK11195, Ro4-54864, PIGAs) on glioma C6 cell steroid synthesis. (**A**) Dose-dependent effects of the compounds with longest RTs (>40 min) on C6 steroidogenesis (**B**) Dose-dependent effects of the compounds with shortest RTs (<40 min) on C6 steroidogenesis. C6 cells were cultured with increasing concentrations of ligands (0–100 μM) for 2 h in serum-free media and pregnenolone released into the media was assessed by ELISA. The results are expressed as the mean of three separate experiments, and data were fitted with a dose–response curve with Hill slope of 1.0.

The steroidogenic efficacy of TSPO ligands was measured in terms of pregnenolone production in C6 glioma cells following exposure with increasing ligand concentrations for a fixed incubation time. For each TSPO ligand, potency (EC_50_ value) was derived by sigmoidal concentration-dependent curve and efficacy (E_max_ value, relative to the highest tested concentration of TSPO ligand) was calculated with respect to control (DMSO-treated sample), corresponding to basal pregnenolone production. In Fig. 4, the curves of TSPO ligand-stimulated pregnenolone production are shown. The EC_50_ and E_max_ values are detailed in Table 3. Specifically, among all tested TSPO ligands, M-PIGA, PIGA823 and PIGA1138 had the highest efficacy. The majority of the tested TSPO ligands showed efficacy to stimulate pregnenolone production ranging from 140% up to 179% (basal value was set to 100%).

### Correlation between RT and steroidogenesis efficacy

**Figure 5:**
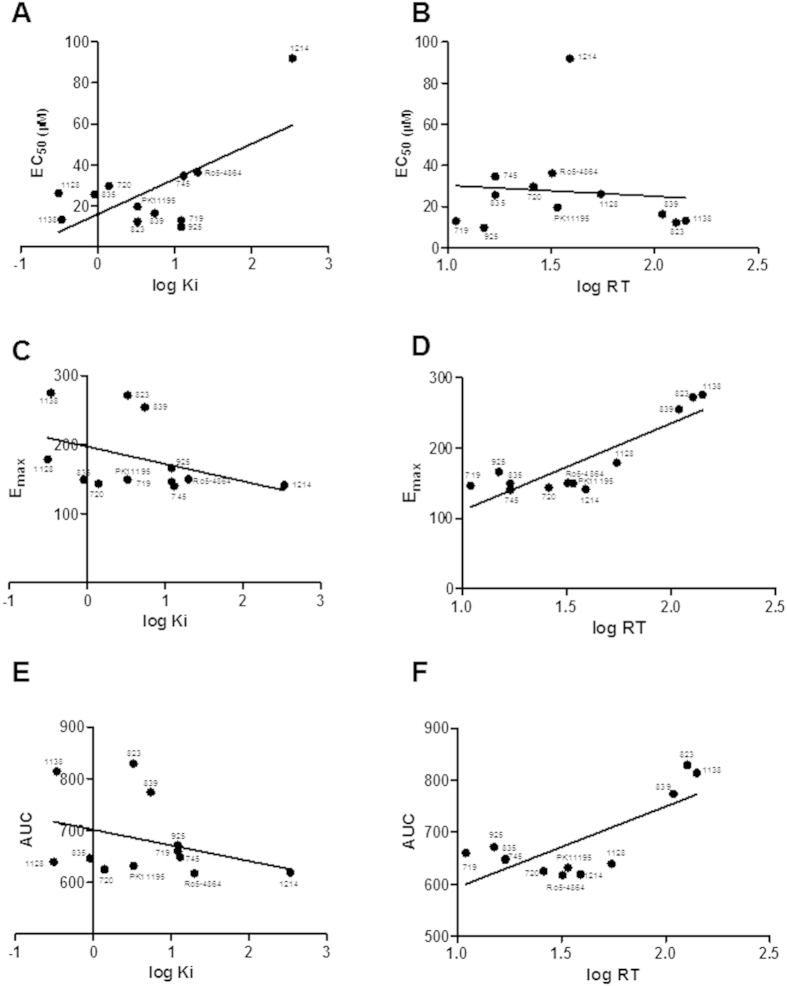
Correlation analyses between kinetic/thermodynamic and steroidogenic parameters. TSPO compound names were included next to their respective data points (for PIGA compounds only the ID numbers were shown). (**A**) Scatter plot of the EC_50_ values against thermodynamic parameters (logK_i_) of test TSPO ligands; (**B**) Scatter plot of the EC_50_ values against kinetic parameters (logRT) of test TSPO ligands; (**C**) Scatter plot of the E_max_ values against thermodynamic parameters (logK_i_) of test TSPO ligands; (**D**) Scatter plot of the E_max_ values against kinetic parameters (logRT) of test TSPO ligands; (**E**) Scatter plot of the AUC values against thermodynamic parameters (logK_i_) of test TSPO ligands; (**F**) Scatter plot of the AUC values against kinetic parameters (logRT) of test TSPO ligands.

In Fig. 5, correlation analyses are reported between the TSPO ligand-mediated steroidogenic potency or efficacy and either binding affinity (K_i_) or Residence Time (RT). The steroidogenic potency of TSPO ligands correlated with the logarithm of K_i_ (Pearson r = 0.6553; P = 0.0207; r^2^ = 0.4295) (Fig. 5A), in agreement with previous reported data^40^,^41^,^42^, but not with RT (Pearson r = 0.08962; P = 0.7818; r^2^ = 0.0803) (Fig. 5B). The steroidogenic efficacy of TSPO ligands did not significantly correlate with K_i_ (Pearson r = −0.3489; P = 0.2663; r^2^ = 0.1217) (Fig. 5C). Conversely, a highly significant correlation was observed between steroidogenic efficacy and RT (Pearson r = 0.8526; P = 0.0004; r^2^ = 0.7270) (Fig. 5D). Correlation analyses were also performed between the logarithm of K_i_ or RT and the area under the dose-response curve (AUC), a value that combines potency and efficacy of a drug into a single parameter^43^. When the relationship between AUC and the logarithm of K_i_ was analyzed, no correlation was found (Pearson r = −0.3308; P = 0.2937; r^2^ = 0.1094) (Fig. 5E). On the contrary, the AUC values significantly correlates with logarithm of RT (Pearson r = 0.7563; P = 0.0044; r^2^ = 0.5720) (Fig. 5F).

## Discussion

The TSPO drug discovery has followed the traditional thermodynamic equilibrium constant (K_d_ or K_i_) paradigm to identify lead compounds. However, binding kinetics parameters, especially the Residence Time of a drug on its target, are becoming critical predictors for *in vivo* drug efficacy^25^,^26^. In the present manuscript, accurate kinetic parameters for TSPO ligands were obtained by the use of the mathematical model of Motulsky and Mahan^32^ that has been shown to be highly accurate in determining the binding kinetics of unlabeled ligands at several targets^33^,^34^,^35^. This mathematical model has overcame the high cost of compound radiolabeling in order to measure directly the association and dissociation rates of a drug to the target^44^. For binding kinetics determination, classical TSPO ligands (the isoquinoline PK11195 and the benzodiazepine Ro5-4864) and the TSPO ligand PIGAs^27^ (Table 1) were selected. Specifically, the majority of the tested TSPO compounds resulted rapid dissociating competitors of [^3^H]PK11195 binding sites (PIGA719, PIGA720, PIGA745, PIGA835, PIGA925, PIGA1124 and Ro5-4864) (Table 2). Conversely, the compound PIGA823, PIGA839 (M-PIGA), PIGA1128, and PIGA1138 resulted slow dissociating competitors (Table 2). Concerning the molecular determinants underling the TSPO-ligand kinetic interactions, no literature data are available. The estimation of the RT for a large number of TSPO ligands could support in determining new Structure Activity Relationship (SAR) based on the ligand kinetic parameters. Indeed up to date, SAR has been rationalized only on binding affinity parameters using pharmacophore hypotheses^27^,^28^,^45^,^46^ and a 3D model^29^ based on the newly published NMR structure of mouse TSPO (PDB code 2MGY)^47^. Notably, the steroidogenic efficacy of TSPO ligands did not correlated with the compound binding affinity^21^, suggesting the limitation of a SAR affinity-based strategy.

Herein, a highly significant positive correlation between the efficacy to stimulate *in vitro* steroidogenesis of TSPO ligands and their kinetic parameter RT was found. Remarkably, the efficacy did not correlate with the thermodynamic equilibrium constant K_i_ of the TSPO ligands. Taken together, these results indicate that the key factor for robust steroidogenic TSPO ligand efficacy is not the binding affinity *per se*, but rather the time the compound spends on the target. Such this novel concept for TSPO ligands suggests the importance of using the RT parameter rather than the constant K_i_ during the *in vitro* characterization of TSPO compounds in relation to their steroidogenic activity.

The results discussed thus far allow us to outline a preliminary efficacy-based SAR for the interaction of PIGAs with TSPO. Data in Table 2 suggest a cooperative effect between the size of the substituents on the amide nitrogen and the lipophilicity of the aryl group at the 2-indole position. In particular, high retention times and high efficacy seem to derive from the presence of an highly lipophilic moiety at the 2-position (C_6_H_4_-4-CH_3_, C_6_H_4_-4-Cl, naphth-2-yl), combined with at least one of the two *N*-alkyl groups with a number of carbon atoms in the 1–3 range (PIGA839, PIGA823, PIGA1138).

From a therapeutic perspective, this new concept paves the way to identify TSPO drugs with promising pharmacological activities, including anxiolytic effects. Scientific community has directed particular interest to TSPO ligands for the treatment of anxiety-related disorders, as they have shown fast-acting anxiolytic properties without the typical side-effects of benzodiazepine-based regimes^15^,^48^,^49^. Differently to benzodiazepines, which act as direct modulators of the GABA_A_ receptor, TSPO ligands generally enhance GABAergic neurotransmission via the promotion of neurosteroidogenesis without direct effects at the GABA_A_ receptor^17^. Neurosteroids, especially the 3-alpha-reduced steroids, are potent positive allosteric modulators of GABA_A_ receptor^17^. Consistent with such evidences, previous our data^30^ have shown that the medium from PIGA839 (M-PIGA)-treated human glial cell model contained high levels of allopregnanolone, one of the major positive GABA_A_ receptor allosteric modulator. The conditioned medium potentiated the ^36^Cl^−^ uptake into cerebral cortical synaptoneurosomes, suggesting a positive modulation of GABA_A_ receptor activity^30^. The PIGA ligands have been already tested for their potential *in vivo* anxiolytic effects by means of the Elevated Plus-Maze (EPM) paradigm in the rat (PIGA823: compound number 32 in^28^, and PIGA839 (M-PIGA) in^30^). In the EPM test, either PIGA823 and PIGA839 (M-PIGA), characterized by long RT and high steroidogenic efficacy, have elicited a significant anxiolytic activity (PIGA823: RT = 127 min, E_max_ pregnenolone = 272%; PIGA839 (M-PIGA): RT = 109 min; E_max_ pregnenolone = 254%). Conversely, it has been documented that the classical TSPO ligand PK11195 and Ro5-4864, which are characterized by shorter RT and lower pregnenolone E_max_ (PK11195: RT = 34 min, E_max_ pregnenolone = 153%; Ro5-4864: RT = 32 min; E_max_ pregnenolone = 150%) than PIGA823 and PIGA839 (M-PIGA), does not determine anxiolytic effect in EPM test^50^. In addition, PK11195 has been used to antagonize anxiolytic effects exerted by other TSPO ligands (high steroidogenic efficacy, such as FGIN-1-27, FGIN-1-44 and YL-IPA08)^4^,^6^,^19^. These retrospective assessments suggested that a long RT predicts the anxiolytic activity of a TSPO ligand. Moreover, RT is a better efficacy predictive measure than the thermodynamic equilibrium constant K_d_ or K_i_. Indeed, PK11195, PIGA823 and PIGA839 (M-PIGA) showed comparable K_i_ values (PK11195: K_i_ = 3.60 nM; PIGA823 K_i_ = 3.30 nM; PIGA839 (M-PIGA) K_i_ = 5.50 nM), irrespective to their *in vivo* activity. Consistent with our data, it has been recently demonstrated discrepancy between the K_i_ of known anxiolytic TSPO ligands (Etifoxine and XBD173) and the enhancement of neurosteroid synthesis^21^. Specifically, Etifoxine, which is already clinically approved for the treatment of anxiety-related disorders, is more potent to stimulate neurosteroidogenesis than XBD173, although its binding affinity to TSPO was approximately 140 fold lower than XBD173. These findings have suggested that the efficacy of such TSPO ligands to stimulate neurosteroid synthesis, thereby leading to anxiolytic effects, cannot be concluded from their binding affinity to TSPO.

In conclusion, the present results indicate that the Residence Time is a better parameter to estimate the steroidogenic effectiveness of a TSPO ligand compared to the equilibrium thermodynamic parameters, corroborating the importance of the drug-target interaction dynamics in predicting the drug efficacy. These findings, in combination with the future availability of a RT database, open the way to optimize TSPO ligands as promising therapeutic tools.

## Materials and Methods

### Reagents

[^3^H]PK11195 (Specific Activity, 85.7 μCi/nmol) and Ultima Gold scintillation mixture were obtained from Perkin-Elmer Life Sciences. PK11195, protease inhibitors and GF/C glass fiber filters were purchased from Sigma-Aldrich. 2-arylindol-3-ylglyoxyl derivatives were synthesized as previously described^27^,^28^,^29^. Dulbecco’s modified Eagle’s medium, fetal bovine serum, L-glutamine, penicillin, and streptomycin were from Lonza (Milano, Italy). Enzyme immunoassay (ELISA) for pregnenolone measurement was obtained from IBL (Hamburg, Germany). SU10603 and trilostane were gifts from Novartis Farma (Varese, Italy) and Dr. Zister (University of Dublin, Dublin, Ireland), respectively. Protein assay reagent was obtained by Bio-Rad Laboratories Inc. All other chemical reagents were obtained by commercial sources.

### [^3^H]PK11195 binding saturation assays

Membranes from rat kidneys were prepared as described previously^51^. All the experimental procedures were carried out following the guidelines of the European Community Council Directive 86–609 and have been approved by the Committee for animal experimentation of the University of Pisa. The resulting membrane pellets were aliquoted and frozen at –20 °C. For all radioligand binding assays, an aliquot of membranes was thawed, suspended in assay buffer (AB, Tris-HCl 50 mM, pH 7.4) and homogenized using Ultraturrax. In cell membrane homogenate, protein content was measured by the Bradford method^52^ using the Bio-Rad Protein Assay reagent.

Membrane homogenates (30 μg of proteins) were incubated with increasing [^3^H]PK11195 concentrations (0.1–20 nM; Specific Activity, 85.7 μCi/nmol) in the final volume of 500 μl of AB for 90 min at 0 °C. Non-specific [^3^H]PK11195 binding was obtained in the presence of 1 μM PK11195 (solubilized with ethanol); the solvent concentration was less than 1% and did not interfere with specific [^3^H]PK11195 binding. After incubation time, samples were filtered rapidly under vacuum through GF/C glass fiber filters. After being washed three times with 3 ml of AB, radioactivity trapped on the filter was measured by liquid scintillation counter (TopCount; PerkinElmer Life and Analytical Sciences; 65% counting efficiency).

### PIGA TSPO ligands

The compounds PIGA719, PIGA720, PIGA745, PIGA835, PIGA839 (M-PIGA)^27^, PIGA925, PIGA823, PIGA922^28^, PIGA1214, PIGA1128, PIGA1138 were synthesized following experimental procedure previously described by us^29^.

### [^3^H]PK11195 binding displacement assays

Membrane homogenates (20 μg of proteins) were incubated with increasing concentrations of unlabeled TSPO ligand and 1 nM [^3^H]PK11195 (Specific Activity, 85.7 μCi/nmol) in the same above described conditions. The inhibitory constant (K_i_) determination was performed for PK11195 and additional compounds, including Ro5-4864 (Sigma-Aldrich Milano, Italy) and PIGA ligands^27^,^28^,^29^.

### [^3^H]PK11195 binding ‘traditional’ kinetics assays

The [^3^H]PK11195 k_off_ was determined by incubating membrane homogenates (30 μg of protein) with [^3^H]PK11195 at one fixed concentration, corresponding to approximately ten-fold its K_d_ value, in a final volume of 500 μl AB at 0 °C. To obtain [^3^H]PK11195 concentration, corresponding to 10 × K_d_, approximately 1.5 μCi of [^3^H] had to be added per assay. Since 1 μCi of [^3^H] per assay represents the practical upper limit of radiotracer usage, the radioligand was diluted with unlabeled PK11195 to reduce its specific activity. In brief, the specific activity of [^3^H]PK11195 was reduced to ¼ of its initial original specific activity (Specific Activity, 21.4 μCi/nmol). This allowed us to use a maximum amount of approximately 0.38 μCi of [^3^H] per sample. After a pre-incubation of 2 h, the dissociation was initiated by addition of 5 μM PK11195. The amount of radioligand bound to TSPO was measured at various time intervals for a total duration of 2 h.

To determine [^3^H]PK11195 k_on_, the observed association rate constant (k_ob_) was calculated at different concentrations of [^3^H]PK11195 (Specific Activity, 21.4 μCi/nmol). The experiment was initiated (t = 0) by addition of [^3^H]PK11195 to membrane homogenates (30 μg of proteins) in a final volume of 500 μl of AB and incubated up to 2 h. To establish whether [^3^H]PK11195 binding was stable for longer times than 2 h, incubation times were prolonged up to 5 h in some kinetic association binding assays. Free [^3^H]PK11195 was separated at multiple time points to construct association kinetic curves. Incubations for both the [^3^H]PK11195 dissociation and association assays were terminated and samples were obtained as above described.

### Unlabeled TSPO ligand competition kinetic association assays

The unlabeled TSPO ligand kinetic parameters were assessed using the theoretical model of Motulsky and Mahan^32^. Unlike methods in which one compound is pre-equilibrated with the receptor, this approach involves the simultaneous addition of both radioligand and competitor to receptor preparation, so that at t = 0 all receptors are unoccupied. [^3^H]PK11195 (approximately 30 nM; SA, 21.4 μCi/nmol) was added simultaneously with unlabeled compound to membrane homogenates (30 μg of proteins) in a final volume of 500 μl AB. The degree of bound to TSPO was assessed at multiple time points by filtration harvesting and liquid scintillation counting, as above described. The assay was performed using concentration of PK11195 corresponding to one-, three- and ten-fold its K_i_. For ‘simplified’ competition kinetic association assays, the experiments were performed using concentration of unlabeled TSPO ligands corresponding to three-fold their K_i_.

### Pregnenolone measurement

Pregnenolone assessment was performed using rat C6 glioma cells as an *in vitro* steroidogenic model, as previously described^29^. C6 cells were cultured in Dulbecco’s modified Eagle’s medium supplemented with 10% fetal bovine serum, 2 M L-glutamine, penicillin at 100 U/mL, and streptomycin at 100 μg/mL. Cell cultures were maintained in a humidified atmosphere of 5% CO_2_ and 95% air at 37 °C. Before the measurement of pregnenolone production, the cells (seeded in 96-well plates at a density of ∼10^5^ cells/well) were washed 2 times with a salt medium, consisting of 140 mM NaCl, 5 mM KCl, 1.8 mM CaCl_2_, 1 mM MgSO_4_, 10 mM glucose, and 10 mM *N*-2-hydroxyethylpiperazine-*N*′-2-ethanesulfonic acid(HEPES)–NaOH (pH 7.4) plus 0.1% bovine serum albumin. For the measurement of pregnenolone secreted into the medium, the further metabolism of pregnenolone was blocked by the addition of trilostane (25 μM) and SU10603 (10 μM) (inhibitors of 3β-hydroxysteroid dehydrogenase and 17α-hydroxylase, respectively) to the salt medium. The addition of PK11195, Ro5-4864 or PIGAs to the C6 cells was accomplished by complete change of the salt medium to a medium containing increasing concentrations of the compounds (ranging from 0 to 100 μM). The final concentration of vehicle (DMSO or ethanol) was constant for all of the samples and did not exceed 0.5% (v/v), a concentration that did not affect steroid production on its own. At the end of the incubation periods (2 hours), the cell medium was collected and the amount of pregnenolone secreted into the medium was quantified by an enzyme immunoassay, under the conditions recommended by the supplier. Cross-reactivity with other steroids was typically less than 1% and cross-reactivity with progesterone is 6%. The sensitivity of the assay was 0.05 ng/ml. Unknown samples were compared with concurrently run standards of pregnenolone using a one-site competition model (calibrator curve).

### Data analysis

All experiments were analyzed by either linear or non linear regression using Prism 5.0 (GraphPad Software Inc., San Diego, CA). Equilibrium K_d_ and maximum binding sites (B_max_) values of [^3^H]PK11195 at TSPO were obtained by computational analysis of saturation curves. Competition displacement binding were fitted to sigmoidal (variable slope) curves. The concentration of test compounds that inhibited [^3^H]PK11195 binding to kidney membranes by 50% (IC_50_ values) obtained from the inhibition curves were converted to K_i_ values using the method of Cheng and Prusoff^53^. [^3^H]PK11195 dissociation data were fitted to a one-phase exponential decay function and the t_1/2_ value obtained was transformed into a k_off_ rate using the Equation:


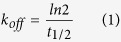


[^3^H]PK11195 association data were fitted to a single phase exponential association function to calculate an observed rate constant k_ob_.

Association and dissociation rates for unlabeled TSPO ligands were calculated by fitting the data in the competition association model using the ‘kinetics of competitive binding’ assay, defining the amount of radioligand bound to receptor ([RL]) as a function of time^32^:


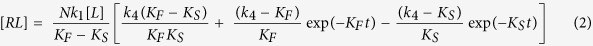



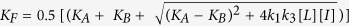



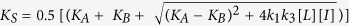



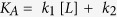



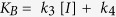


The abbreviations used are: [RL] is the receptor-radioligand complex, as the specific [^3^H]PK11195 binding (fmol/mg of protein); [L] is the concentration of [^3^H]PK11195 used (nM), [I] is the concentration of unlabeled ligand (nM); t is the time in min; N is the total concentration of TSPO (fmol/mg of protein); k_1_ and k_2_ are the k_on_ (M^−1^ min^−1^) and k_off_ (min^−1^) of [^3^H]PK11195, respectively, determined from the radioligand association assay; k_3_ is the k_on_ value (M^−1^ min^−1^) of the unlabeled ligand; k_4_ is the k_off_ value (min^−1^) of the unlabeled ligand.

Correlation analyses were performed by Pearson correlation.

## Additional Information

**How to cite this article**: Costa, B. *et al.* TSPO ligand residence time: a new parameter to predict compound neurosteroidogenic efficacy. *Sci. Rep.* **6**, 18164; doi: 10.1038/srep18164 (2016).

## Figures and Tables

**Table 1:**
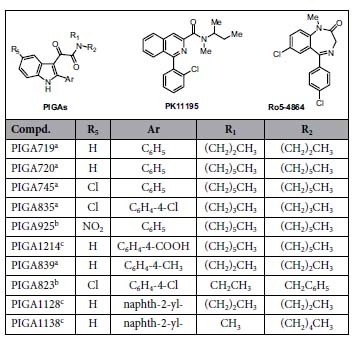
Structures of PIGAs, PK11195, and Ro5-4864.

^a^Primofiore *et al.*, 2004.

^b^DaSettimo *et al.*, 2008.

^c^Barresi *et al.*, 2015.

**Table 2:** TSPO ligand binding affinity and kinetic parameters obtained by equilibrium binding and kinetic association assays.

TSPO ligand	Equilibrium K_i_ orKd (nM)	K_on_ (M^−1^. min^−1^)	K_off_ (min^−1^)	Kinetically-derivedK_d_ = K_off_/K_on_ (nM)	RT = 1/K_off_(min)
PK11195 (Saturation binding)^a^	3.60 ± 0.41	NA	NA	NA	NA
PK11195 (Displacement)^b^	3.39 ± 0.34	NA	NA	NA	NA
PK11195 (Association and dissociation)^c^		8.30 ± 0.64 × 10^6^	0.030 ± 0.002	3.61 ± 0.21	33 ± 4
PK11195 (Standard competition association)^d^		9.20 ± 0.71 × 10^6^	0.034 ± 0.004	3.70 ± 0.43	29 ± 3
PK11195 (Simplified competition assay)^e^		9.30 ± 0.94 × 10^6^	0.029 ± 0.003	3.12 ± 0.37	34 ± 3
Ro5-4864	20.04 ± 2.36	1.29 ± 0.16 × 10^6^	0.031 ± 0.002	24.04 ± 2.01	32 ± 3
PIGA719	12.24 ± 1.1	7.89 ± 0.62 × 10^6^	0.094 ± 0.007	11.95 ± 1.00	11 ± 2
PIGA720	1.40 ± 0.28	2.18 ± 0.29 × 10^7^	0.039 ± 0.004	1.79 ± 0.24	26 ± 2
PIGA745	13.14 ± 1.16	3.84 ± 0.51 × 10^6^	0.058 ± 0.003	15.12 ± 1.16	17 ± 1
PIGA823	3.30 ± 0.31	4.30 ± 0.33 × 10^6^	0.008 ± 0.001	1.86 ± 0.17	127 ± 4
PIGA835	0.91 ± 0.11	5.78 ± 0.56 × 10^7^	0.058 ± 0.005	1.00 ± 0.19	17 ± 1
PIGA839 (M-PIGA)	5.50 ± 0.47	1.69 ± 0.27 × 10^6^	0.009 ± 0.001	5.45 ± 0.60	109 ± 4
PIGA925	12.23 ± 3.14	6.78 ± 0.42 × 10^6^	0.068 ± 0.004	10.01 ± 1.13	15 ± 2
PIGA1128	0.31 ± 0.02	8.10 ± 0.36 × 10^7^	0.018 ± 0.001	0.22 ± 0.05	55 ± 2
PIGA1138	0.34 ± 0.03	4.30 ± 0.30 × 10^7^	0.007 ± 0.001	0.17 ± 0.03	141 ± 4
PIGA1214	343.01 ± 15.94	9.05 ± 0.51 × 10^4^	0.030 ± 0.004	332.05 ± 15.04	39 ± 2

Values are means ± SEM of three experiments performed in duplicate. NA = not applicable.

^a^[^3^H]PK11195 binding performed using increasing concentration of radioligand.

^b^displacement of [^3^H]PK11195 binding using increasing concentration of PK11195.

^c^the binding kinetics of [^3^H]PK11195 were determined by ‘traditional’ association and dissociation assays.

^d^the binding kinetics of unlabeled PK11195 were determined by adding a concentration equivalent to one-, three- and ten-fold the K_i_ value of PK11195.

^e^the binding kinetics of unlabeled PK11195 were determined by adding a concentration equivalent to only three-fold the K_i_ value of PK11195.

**Table 3:** Experimental kinetic/thermodynamic data and steroidogenic parameters for TSPO ligands.

TSPO ligand	EC_50_(μM)	Emax (at 100 μM)(vehicle set to 100%)	Equilibrium K_i_ or Kd(nM)	RT (min)
PK11195	19.5 ± 1.8	153 ± 4	3.30 ± 0.34	34 ± 3
Ro5-4864	36.2 ± 2.5	150 ± 4	20.04 ± 2.36	32 ± 3
PIGA719	12.9 ± 1.5	146 ± 2	12.24 ± 1.1	11 ± 2
PIGA720	29.7 ± 3.1	144 ± 4	1.40 ± 0.28	26 ± 2
PIGA745	34.6 ± 3.6	140 ± 5	13.14 ± 1.16	17 ± 1
PIGA823	12.2 ± 1.3	272 ± 11	3.30 ± 0.31	127 ± 4
PIGA835	25.6 ± 2.3	149 ± 4	0.91 ± 0.11	17 ± 1
PIGA839 (M-PIGA)	16.3 ± 1.4	254 ± 5	5.50 ± 0.47	109 ± 4
PIGA925	9.71 ± 1.0	166 ± 5	12.23 ± 3.14	15 ± 2
PIGA1128	26.1 ± 1.5	179 ± 7	0.31 ± 0.02	55 ± 2
PIGA1138	13.1 ± 1.4	275 ± 5	0.34 ± 0.03	141 ± 4
PIGA1214	92.0 ± 5.6	141 ± 4	343.01 ± 15.94	39 ± 2

Values are means ± SEM of three experiments performed in duplicate. Potency (EC_50_ value) of TSPO ligands was derived by sigmoidal concentration-dependent curves. Efficacy (E_max_) corresponds to amount of pregnenolone production by 100 μM TSPO ligand concentration.
